# Awareness, use, attitude and perceived need for Complementary and Alternative Medicine (CAM) education among undergraduate pharmacy students in Sierra Leone: a descriptive cross-sectional survey

**DOI:** 10.1186/1472-6882-14-438

**Published:** 2014-11-08

**Authors:** Peter B James, Abdulai J Bah

**Affiliations:** Department of Pharmacognosy and Phytochemistry, Faculty of Pharmaceutical Sciences, College of Medicine and Allied Health Sciences, University of Sierra Leone, Freetown, Sierra Leone; Department of Pharmacology Faculty of Basic Medical Sciences, College of Medicine and Allied Health Sciences, University of Sierra Leone, Freetown, Sierra Leone; Complementary and Alternative Medicine Department, Pharmacy Board of Sierra Leone, Medical Stores, Compound New England Ville, Freetown, Sierra Leone

**Keywords:** Complementary and alternative medicine, Pharmacy students, Awareness, Attitude, Use, Education, Sierra Leone

## Abstract

**Background:**

The widespread use of CAM around the world requires health professionals including pharmacists to have the required knowledge to better advise their patients. This has lead to an increased need for the inclusion of CAM instruction into the mainstream undergraduate Pharmacy education. This study was designed to describe pharmacy students awareness, use, attitude and perceived need for CAM education at COMAHS-USL and at the same time, determine how these descriptive outcomes are influenced by the socio-demographic variables considered in this study.

**Methods:**

A descriptive cross-sectional survey was conducted among undergraduate pharmacy students (n = 90) at the College of Medicine and Allied Health Sciences, University of Sierra Leone using a structured questionnaire. Chi square, fisher exact test, and general linear model univariate analysis were used to compare data between independent cohorts.

**Results:**

All 90 (100%) of the students were aware and have used (except Ayurveda) at least one of the listed CAM modalities. Herbal/Botanical/Supplements followed by Spirituality/Prayer were the most commonly known and used CAM modalities. Almost two thirds of students considered the CAM modalities they have used to be effective and not harmful. Overall, pharmacy students had a positive attitude towards CAM (Mean attitudinal score = 34.9 ± 4. 5 (range 19–43)) with fourth and fifth year students showing a significantly less positive attitude as compared to the first, second and third year (B = −3.203 p = 0.001, 95% confidence interval - 5.093 to −1.314). The media [53 (58.9%)] was the most frequent source of information about CAM. Nearly all students [89 (98.9%)] agreed that CAM knowledge is important to them as future pharmacist and that CAM should be included into the Pharmacy curriculum at COMAHS-USL [81 (90.0%)].

**Conclusion:**

Pharmacy students in Sierra Leone are aware of and have used at least one of the CAM modalities and do show a positive attitude towards CAM. This was demonstrated by their overwhelming endorsement for CAM course to be part of the undergraduate pharmacy training at COMAHS-USL. This study among others will inform and guide the development and implementation of CAM instruction at COMAHS-USL.

**Electronic supplementary material:**

The online version of this article (doi:10.1186/1472-6882-14-438) contains supplementary material, which is available to authorized users.

## Background

Since time immemorial, mankind has developed unique indigenous health systems, practices, and products which are outside conventional scientific medicine collectively known as Complementary and Alternative Medicine (CAM) [1]. Complementary practices are healthcare interventions that are used together with conventional medical practice whilst alternative health practices are considered to be an option to conventional medical practice [2]. This form of health care is greatly influenced by culture and tradition of society and is known to play a great role in the delivery of health care in many countries around the world [1]. CAM encompasses natural products (herbs, vitamins and probiotics), mind and body practices (acupuncture, massage therapy, Chai chi etc.) and other traditional medical practices such African traditional medicine, traditional chinese medicine in China and Ayurvedic medicine in India [2]. The use of Complementary and Alternative Medicine (CAM) has increased dramatically in both the developed and developing world due to its accessibility, affordability as well as its perceived efficacy and safety in treating diseases as compared to allopathic medicine. For instance, in Italy, Germany, Canada, and France the percentage of CAM use within their populations range from 70% to 90% [3]. In the African region, 70–95% of its population rely on traditional healing methods, including herbal remedies, for maintenance of health and wellbeing [4].

In Sierra Leone, even though conventional medical practice is the main form of health care, CAM especially traditional medicine, still enjoys widespread popularity and usage among the population. Anecdotal evidence suggests at least 70% of the population use CAM of which biological based therapies are the most common. In response to the Beijing declaration [5] and the WHO Regional committee for Africa resolution AFR /RC50 /R3 [6], a national traditional medicine policy was developed that serves to promote traditional medicine development and integration into the health system as well as promoting its rational use among health service providers [7]. Achieving this goal requires health professionals to be well knowledgeable about CAM practices and products with regards to their quality, effectiveness and safety and so be better equipped to advise patients.

In most part of the world, pharmacists are trusted healthcare providers and at the forefront of patient therapy. They are known to provide appropriate, reliable information and advice to patients with regards to the safe and effective use of their medicines [8, 9]. However, anecdotal evidence suggests that most pharmacists and other health professionals in the mainstream health care service delivery in Sierra Leone have little or no knowledge about CAM and are usually hesitant to respond to patient concerns regarding the use of CAM. This has also been the conclusion of various studies elsewhere [10–14]. For instance, a survey of community pharmacists in Australia revealed that less than 15% of them were comfortable in responding to queries regarding the safety, benefits and interaction of CAM. They also indicated that lack of formal training is one of the limitations in providing correct information about CAM [10]. This has the potential to put patients at risk and may lead to patient exposure to interactions with conventional medicines or adverse effects. Therefore, it is imperative that health professionals are knowledgeable about the quality, effectiveness and safety of CAM. Implementing programs that seek to address the knowledge gap about CAM among Pharmacists and other health professionals is important to achieving the overall vision of CAM integration into the national health care system.

The successful inclusion of CAM into the health care system requires that it is included into the health professionals educational system. This will allow pharmacist and other health professionals to be formally train and be in a better position to advise patients on CAM use. However, the willingness of students to learn about CAM may be influenced by certain determinants like their previous knowledge and experience as well as their attitude toward CAM. Research conducted in western countries that assessed medical students, doctors, pharmacy students, pharmacists and nurses attitudes and perceptions regarding CAM indicated that medical and pharmacy students show an increased interest and positive attitude towards CAM and a need for it to be uncluded into pharmacy and medical undergraduate curricula [15–23]. In response to this, CAM has been incorporated into mainstream medical and pharmacy curricula in most medical and pharmacy schools in the developed world [24–26]. For instance, in the USA, 64% of medical colleges and 70% to 85% of pharmacy schools and colleges do offer CAM courses [27, 28].

In the past decade, some countries in the Africa region have included traditional medicine in health professional training curricula. For example, countries such as Ghana, BurkinaFaso and Nigeria have developed degree and diploma courses to train medical herbalists and CAM practitioners while some universities in South Africa have introduced short courses in traditional medicine in their pharmacy curricula [29]. In Sierra Leone, the Pharmacy faculty of COMAHS-USL offers courses on herbal medicine at the fourth year level. A more comprehensive module that includes other CAM modalities preferably integrated within the existing pharmacognosy course, is needed. Notwithstanding, such developments, research regarding CAM in Africa has focused on the assessment of knowledge, attitude, perception, and use among hypertensive, diabetes, cancer, HIV/AIDS patients and the general healthy public [30–36]. However, there is still a paucity of data regarding pharmacy and medical students’ or health providers’ knowledge, attitude and perception about CAM in the African region. In Sierra Leone, no such research has been done so far regarding pharmacist perception, attitude and use of CAM. To address this gap, we decided to carry out a study that aims to describe pharmacy students awareness, use, attitude and perceived need for CAM education at COMAHS-USL and at the same time, determine how these descriptive outcomes are influenced by the socio-demographic variables considered in this study.

## Methods

### Study design and population

A descriptive cross-sectional survey was conducted among first to fifth year pharmacy students at the College of Medicine and Allied Health Sciences, University of Sierra Leone (COMAHS-USL). Ethical approval of this study was obtained from the Research and Ethics Committee at COMAHS-USL. This study was conducted between January 2014 and March 2014. COMAHS-USL is the only tertiary institution in Sierra Leone that offers an undergraduate pharmacy program. Students are accepted to the five year Bachelor of pharmacy with Honours (B. Pharm. Hons) program either directly from senior high school or from a specific premedical course. There is no formal teaching of CAM in the curriculum at COMAHS-USL although pharmacy students take courses in pharmacology, pharmacognosy and Phytochemistry in the third and fourth years respectively and some CAM subjects are covered within them. At the time of the survey, ninety nine (99) pharmacy students were enrolled at the university and this constitutes the study population.

### Study questionnaire

The questionnaire composed of 4 sections. Section A consists of questions on demographics of respondents (age, sex, year of study and religion). Section B asked pharmacy students about awareness, use, effectiveness, and harmfulness of the various CAM modalities. Respondents awareness and use were assessed using a ‘Yes’ or ‘No’ option. If a student answered ‘Yes’, to use of CAM modality/ies, he/she was to answer further questions relating to the perceived effectiveness and harmfulness. Effectiveness was measured on a five point likert type scale. The point descriptors were very ineffective, ineffective, neutral, effective, very effective. With regards to harmfulness, students were also asked to indicate their views on a five-point likert type scale. The point descriptors were very harmful, harmful, neutral, not harmful, very not harmful. Section C looks at respondents general attitudes toward CAM using a previously validated 10 item CAM health belief questionnaire (CHBQ) [37] as a template. This CHBQ was adapted to fit our own setting. The 10 different CHBQ statements were designed with a five-point likert type rating scale. The point descriptors were strongly disagree, disagree, neutral, agree, strongly agree of which each were scored (where 1 = strongly disagree and 5 = strongly agree). The total CHBQ score was calculated by adding the scores from the 10 rating statements. The highest CHBQ attitudinal score is 50. As in previous studies [21, 37], a total mean score greater than the hypothetical neutral score of 25 indicated a greater endorsement and a positive attitude. Section D explored sources of CAM information, perceived barriers to CAM implementation in Sierra Leone as well as the perceived need among pharmacy students for CAM education. The questions “Do you think knowledge about CAM is useful to you as a future pharmacy professional” and “Do you think CAM should be included into the Pharmacy undergraduate curriculum at COMAHS” were used to determine pharmacy students perceived need for CAM education. The questionnaire was pretested on 20 pharmacy students to ensure readability, proper design and understanding of its contents. After pretesting, some adjustments were made to make it more comprehensible and simpler to complete. A copy of the CAM survey questionnaire is attached. See Additional file 1.

### Data collection

The questionnaires were distributed to each student together with a written consent form that explained the purpose of the research and assured them of their confidentiality. Signing the consent form indicated that a student was willing to participate in the study. All filled questionnaires were collected by the class leader in each class and returned to the leading investigator.

### Data analysis

Data from completely filled questionnaires were entered into SPSS Package version 16 (SPSS, Inc; Chicago). Descriptive statistics were used to calculate frequency counts and percentages for respondents characteristics. In interpreting the results, all likert scale responses with a degree of agreement were grouped together as positive whilst all responses with a degree of disagreement were considered to be negative. For instance, “very ineffective” and “ineffective” were considered and grouped together as negative response with regards to effectiveness of CAM use. To perform inferential statistics, binary coding was done for all categorical demographic variables i.e. for age group (≤26& >26) Year of study (≤ third year and > third year). Chi square or fisher exact two tailed tests were used to compare the use of the common CAM modalities, student perceived effectiveness and harmfulness as well as student perceived need for CAM education at COMAHS-USL with each of the independent norminal demographic variables. The mean score for the CHBQ items that assessed respondents attitudes were calculated for all respondents. General linear model univariate analysis was employed to compare data between attitudinal score and each independent norminal demographic variable. Differences were considered statistically significant if the p value was less than 0.05.

## Results

### Response rate

A total number of 99 Pharmacy undergraduate students were issued the questionnaires and only 90 completed and returned them given a response rate of 91%.

### Sociodemographic characteristics of respondents

Of the respondents who completed the survey, 17 (18.9%). 18 (20%), 25 (27.8), 16 (17.8) and 14 (15.6%) were from first, second, third, fourth and final year respectively. The frequency distribution based on gender indicated that 64 (71.1%) were males and 26 (28.9%) were females. The age distribution was; ≤20 years 6 (6,7%), 21–26years 61 (67.8%), 27–32years 20 (22.2%) and ≥33 years 3 (3.3%). The frequency distribution with respect to religion indicated that 58 (64.4%) of respondents were Christians and 32 (35.6%) were Muslims. See Table 1.

**Table 1:** **Pharmacy students ( N = 90 ) demographics**

Demographics	n (%)
Year of study	
First year	17(18.9)
Second year	18(20)
Third year	25(27.8)
Fourth year	16(17.8)
Fifth year	14(15.6)
**Sex**	
Male	64(71.1)
female	26(28.9)
**Age group (years)**	
≤20	6(6.7)
21–26	61(67.8)
27–32	20(22.2)
≥33	3(3.3)
**Religion**	
Christianity	58(64.4)
Muslim	32(35.6)

### Respondent awareness and use of CAM

All 90 (100%) of the respondent indicated that they are aware and have used (except Ayurveda) at least one of the listed CAM modalities. With respect to awareness of the individual CAM modalities listed, Herbals/botanicals/supplements was the most commonly known [85 (94.4%)]. This was followed by spirituality/prayer [81 (90%)], massage therapy [72 (80%)]. The least known CAM modality was Ayurveda [10 (11.1%)]. With regards to the use of the individual CAM modalities, again, herbals/botanicals/supplements [63 (70%)] were the most frequently used among pharmacy students. This was followed by spirituality/ prayer [53 (58.9%)] and massage therapy [46 (51.1%)]. All of the respondents [90 (100%) never used Ayurveda. A significant difference was observed between Christians and Muslims with respect to the use of spirituality/prayer as a CAM modality. More Christians used spirituality/prayer as CAM therapy as compared to Muslims (p <0. 001 using chi square test). In addition, among these four frequently used CAM modalities, student gender, age and year of study did not influence their use (p values >0.05). See Table 2.

**Table 2:** **Pattern of awareness and use of CAM modalities among of pharmacy students in Sierra Leone**

CAM modalities	Awareness of CAM n (%)	Self use of CAM n (%)
Acupuncture	52(57.8)	2(2.2)
Herbal/Botanical/Supplements^2^	85 (94.4)	63(70)
Massage Therapy^2^	72(80)	46(51.1)
Ayurveda	10(11.1)	0(0)
Spirituality/Prayer^1^ ^2^	81(90)	53(58.9)
Homeopathy	19(21.1)	1(1.1)
Meditation/Yoga/Relaxation^2^	50(55.6)	22(24.4)

^1^Significant difference (p = 0.000 using chi square test) between Christians and Muslims with respect to the use of spirituality\prayer.

^2^Student gender, year of study, and age group do not significantly (using fisher exact test p > 0.05) influence use of the four common CAM modalities among students.

### Perceived effectiveness and harmfulness of CAM modalities

Respondents were further asked for their views on how effective and harmful the CAM modalities they have used using a likert scale type measurement. With respect to effectiveness, [66 (73.4%)] of respondent indicated that they are effective. In terms of harmfulness], 67 (74.4%)] said they are at not harmful. However, year of the study, religion, age group and sex, did not significant affect student’s perceived effectiveness and harmfulness of the commonly used CAM modalities. See Table 3.

**Table 3:** **Pharmacy students perceived effectiveness and harmfulness of the CAM modalities they have used**

Perceived effectiveness and harmfulness of CAM					
**Effectiveness** ^3^ Likert type	**Very ineffective n (%)**	**Ineffective n (%)**	**Neutral (%)**	**Effective n (%)**	**Very effective n (%)**
n (%)	10(11.1)	9(10)	5(5.6)	50(55.6)	16(17.8)
**Harmfulness** ^3^ Likert type	**Very harmful n (%)**	**Harmful N (%)**	**Neutral n (%)**	**Not harmful n (%)**	**Extremely not harmful n (%)**
n (%)	4(4.4)	9(10)	10(11.1)	49(54.4)	18(20)

^3^Year of study, sex , age group and religion have no significant effect (using Fisher exact two tailed test P > 0.05) on pharmacy student perceived effectiveness and harmfulness of CAM modalities they have used.

### Respondent attitude towards CAM

The CHBQ overall mean score was 34.9 ± 4. 5 (range 19–43). General linear model regression analysis indicated a significant difference (B = −3.203 p = 0.001, 95% confindence interval −5.093 to −1.314) in mean attitudinal scores between the two category of classes with the fourth and fifth year student indicating an average of 3.203 less attitudinal score as compared to the first, second and third year students. See Table 4. However, sex, religion and age group did not significantly affect pharmacy student attitude towards CAM. See Table 4. With respect to individual statements, nearly three fourth (74.5%) of pharmacy students at least agree that patient expectation, health beliefs and values should be integrated into the patient care process whereas, nearly two third (60%) of pharmacy students at least disagree that complementary therapies are a threat to public health. See Table 5.

**Table 4:** **Result of the general linear model univariate analysis to determine the demographic factors that influence attitude toward CAM among pharmacy students in Sierra Leone**

Characteristics	B	SE	p-valve	95% Confidence interval
Year of study				
≤Third year*				
>Third year	−3.203	0.95	0.001	−5.063 to −1.314
**Sex**				
Male*	-			
Female	−1.546	1.02	0.137	−3.560 to 0.495
**Age (years)**				
≤ 26*				
>26	−1.872	2.51	0.459	−6.869 to 3.126
**Religion**				
Christianity*	-			
Muslim	0.335	0.96	0.728	−1.572 to 2.242

*Reference category.

**Table 5:** **Pharmacy students attitude towards CAM**

Statements	SDA n (%)	DA n (%)	N n (%)	A n (%)	SA n (%)
Clinical care should integrate best conventional and CAM practice	2(2.2)	2(2.2)	2(2.2)	51(56.7)	33(36.7)
A patient’s expectations, health beliefs and values should be integrated into the patient care process	6(6.7)	7(7.8)	10(11.1)	42(46.7)	25(27.8)
Complementary therapies include ideas and methods from which conventional medicine could benefit.	2(2.2)	5(5.6)	15(16.7)	51(56.7)	17(18.9)
Treatments not tested in a scientifically recognized manner should be discouraged	14(15.6)	15(16.7)	10(11.1)	17(18.9)	34(37.8)
Complementary therapies are a threat to public health	14(15.6)	40(44.4)	15(16.7)	16(17.8)	5(5.6)
Health and disease are a reflection of balance between positive life-enhancing forces and negative destructive forces	4(4.4)	5(5.6)	26(28.9)	43(47.8)	12(13.3)
Effects of complementary therapies are usually the result of a placebo effect	10(11.1)	16(17.8)	41(45.6)	19(21.1)	4(4.4)
CAM treatment have no true impact on treatment of symptoms, disease conditions	30(33.3)	32(35.6)	20(22.2)	8(8.9)	0(0)
Knowledge of CAM is important to me as a pharmacist	1(1.1)	4(4.4)	4(4.4)	40(44.4)	41(45.6)
Health professional should be able to advise patient on commonly used CAM methods	1(1.1)	3(3.3)	3(3.3)	43(47.8)	40(44.4)

Where SDA = Strongly disagree, DA = Disagree , N = Neutral, A = Agree and SA = Strongly Agree.

### Sources of CAM information

As summarized in Table 6 the common source of CAM information among pharmacy students was the media such as radio, Television, newspapers [53 (58.9%)] followed by CAM practitioners [39 (43.3)] and books [32 (35.6%)]. The least common source of information source was formal CAM training [3 (3.3%)].

**Table 6:** **Sources of CAM Information among Pharmacy student (N = 90)**

Sources	n (%)
Books	32(35.6)
Media	53(58.9)
Journals	20(22.2)
CAM practitioner	39(43.3)
Other health professionals	16(17.8)
Formal CAM training	3(3.3)
Training or apprenticeship with a traditional healer	8(8.9)

### Perceived barriers to appropriate use CAM in Sierra Leone

With regards to perceived barriers to appropriate use CAM in Sierra Leone, pharmacy students indicated that lack of trained professionals [82 (91.1%)] was the key limitation, followed by lack of Knowledge of CAM [79 (87.8%)] and lack of Scientific evidence for practice [77 (85.6%)]. Long time for treatment [45 (50.0%)] was considered to be the least significant barrier. See Table 7.

**Table 7:** **Perceived Barriers to CAM implementation in Sierra Leone**

Perceived barriers	N (%)
Lack of trained professionals	82(91.1)
Lack of Scientific evidence for practice	77(85.6)
Long time for treatment	45(50.0)
Lack of knowledge about CAM	79(87.8)

### Perceived need of pharmacy students for CAM education

When asked about the need for CAM education, nearly all of the pharmacy students [89 (98.9%)] reported that knowledge about CAM will be useful to them as a future pharmacists whilst [81 (90.0%)] agreed that CAM should be included into the undergraduate pharmacy curriculum at College of Medicine and Allied Health Sciences University of Sierra Leone (COMAHS-USL). The overwhelming endorsement by pharmacy student for CAM education was not affected by year of study, student gender, religion and age group. See Table 8.

**Table 8:** **Results of bivariate analysis to determine the association of the sociodemographic variables to the perceived need for CAM education among Pharmacy students (N = 90)**

	Do you think CAM should be included into the Pharmacy curriculum at COMAHS-USL		Chi square	P- value	Do you think knowledge about CAM is useful to you as a future pharmacy professional		Chi square	P-value
	Yes (n)	No (n)			Yes (n)	No (n)		
**Year of study**			2.222	0.262			2.022	0.333
≤Third year	52	8			60	0		
>Third year	29	1			29	1		
**Total**	89	9			89	1		
**Gender**			0.096	0.714			0.411	1.000
Male	58	6			63	1		
Female	23	3			26	0		
**Total**	81	9			89	1		
**Age group (years)**			1.097	0.438			0.347	1.000
≤26	59	8			66	1		
>26	22	1			23	0		
**Total**	81	9			89	1		
**Religion**			0.776	0.482			1.833	0.356
Christianity	51	7			58	0		
Muslim	30	2			31	1		
**Total**	81	9			89	1		

## Discussion

The present study was conducted to evaluate pharmacy students’ awareness, self use, perceived effectiveness and harmfulness of CAM modalities as well as their attitudes toward CAM. Also, sources of CAM information, perceived barriers to CAM implementation and the need for CAM education were looked at. Regarding the awareness and use of the CAM modalities, this study revealed that all of the pharmacy students were aware of at least one of the CAM modalities and have used them (except Ayurveda). This awareness and widespread use of at least one form of CAM has been noted in other studies elsewhere [18, 23, 38, 39] although at different rates. Based on Pharmacy student increased awareness and usage of most of these CAM modalities, it might be argued that it has helped to heightened their curiosity and serve as a motivator to learn more about these modalities. This would have impact their overwhelmining endorsement for CAM to be included into the Pharmacy curriculum. Not surprisingly, the most commonly known and used CAM modality was herbals/botanical/supplements since herbal medicine and nutritional supplements are known to be widely used in Africa [4]. A similar result was observed in a study among Kuwaiti pharmacy and medical students [23] and Bristish undergraduate Pharmacy students [39]. However, studies done among Pakistani [22] and American [17] Pharmacy students, indicated massage therapy to be most widely used CAM modality followed by dietary supplement. Interestingly, spiritual therapy/prayer was the second most commonly known and used CAM modality in this study. This was also observed in a similar study conducted with Kuwaiti pharmacy and medical students [23]. A possible reason for this might be the fact that religion and spirituality are an important aspect of the African culture and tradition, and often do serve as coping mechanisms during ill health [40, 41]. Inferential statistics from this study further showed that the use of spiritual therapy/prayer is significantly influenced by the student religious orientation. Christians students use spiritual therapy/prayer more when compared to muslims. A possible explanation could be that christians do believe in the divine power of healing as compared to muslims and therefore seek this form of complementary therapy when they are ill or experiencing any form of the adversity. The least known and never used CAM modality was Ayurveda. This was expected as this type of CAM practice is not known to be practised in this part of world. With respect to the most commonly used CAM (herbal/botanicals/supplements, massage, spirituality/prayer and meditation/yoga/relaxation) modalities, student gender did not significantly influence their use which is in complete contrast to studies done among pakistani [22] and Czech [21] pharmacy students. However,it was consistent with a study done by Koh and colleaques among pharmacists in Singapore [42]. This differences might be explained by the gender variations in the studied populations.

The safety and efficacy of CAM practices remain largely unknown, advising patients who use or seek alternative treatments should be based on scientific knowledge and experience of use. Nearly three quarters of the students in this study considered the CAM modalities they have used to be effective and not harmful despite limited scientific knowledge. This perception was not influenced by student gender, religion, age and year of study. Although observed in another study [23], this should be a course for concern to educators and health professionals as there is a tendency for them to recommend CAM therapies to patients based on personal experience; a tendency also observed among Pakistani Pharmacy students [22]. This further underscores the need for the introduction of CAM education into the undergraduate pharmacy program at COMAHS-USL.

A positive attitude [overall CHBQ mean attitudinal score 34.9 ± 4. 5 (range 19–43)] toward CAM among Pharmacy students who took part in this study was observed and this resonates with many other studies that assess pharmacy students attitudes toward CAM globally [16, 17, 21, 22, 39, 43]. This finding is also similar to other studies that assess other health professionals attitude towards CAM [19, 20, 44–46]. More importantly, significant variation in positive attitude was observed among the classes of pharmacy students in this study. Students from higher classes (fourth and fifth years) had a less positive attitude (−3.203 p = 0.001, 95% confidence interval - 5.093 to −1.314) than the lower classes (first and second and third years). With respect to their sex, religion and age group, no significant differences were observed. However, a contrasting result was observed among Pakistani and Bristish pharmacy students with regards to sex in which female students tend to have a more positive attitude towards CAM than male students [22, 39]. Although not considered in this study, use of CAM by a friend, family or self can also influence attitude towards CAM as demonstrated among Australian pharmacy students [18]. Pharmacy students’ positive attitude towards CAM in this study was also demonstrated with more than three- quarter of them agreeing that clinical care should integrate the best conventional and CAM practices and nearly two-third were against the notion that Complementary therapies are a threat to public health. To further highlight their positive attitude, nearly all of them [83 (92.2%)] agree that health professionals should be able to advise patients on commonly used CAM methods. As was reported by Pokladnikova and colleaques [21], it is arguable that the previous positive experiences with these CAM modalities might have influenced students’ positive attitudes towards CAM observed in this study. Also, we can speculate that their positive attitude is seen to be translated into an increased need for CAM training at the undergraduate Pharmacy level.

In addition to having positive attitudes, access to evidenced based knowledge about CAM is vital for future Pharmacy professionals when they are required to advise patients who use or seek alternative medical therapy. In this study, the media and CAM practitioners, were the most frequent source of CAM information among students. Most of these sources are misleading and without scientific basis since they are coming from illiterate traditional healers and general public. This finding is in complete contrast to a study done among Pakistani pharmacy students in which professional medical and pharmacy journals were the most common source [22].Lack of trained professionals, lack of knowledge of CAM and scientific evidence for practice were considered as the most common barriers to CAM practice in Sierra Leone. This could serve as key possible reasons for the observed difference between in our setting and that of Pakistan. Similar challenges were also put forward by medical doctors in Lagos Nigeria with regards to traditional medicine practice [46]. This therefore informs the calls for inclusion of evidence based CAM training at the pharmacy undergraduate level; a move widely supported by nearly all of the pharmacy students [81(90.0%)] irrespective of gender, age, level of study and religion. Although not captured in this study, student preferences with regards to the mode of learning, which academic year(s) CAM should be introduced in, as well as faculty members’ attitude and competency to teach CAM modules, are key issues to be considered for the inclusion of CAM into the pharmacy curriculum. In addition, further studies are required to understand the pattern of CAM use among patients and the general public. This will inform the development and implementation of evidence based CAM instruction in the pharmacy faculty at COMAHS-USL. A possible limitation to this study was that student responses were not verified to ensure that their true beliefs and perception about CAM was captured. However, this study does gives a baseline information with regards to the awareness, use and attitude of CAM among pharmacy students in Sierra Leone.

## Conclusion

Pharmacy students in Sierra Leone showed an increased awareness and self reported use of at least one of the CAM modalities considered in this survey. Herbal/botanical/supplement, massage, spirituality/prayer and meditation/yoga/relaxation) were the most commonly known and used CAM modalities among pharmacy student surveyed with herbal/botanical/supplement being the most known and used. These commonly known and used CAM modalities were generally considered to be effective and safe although this perception was not influenced by student gender, age group,religion and year of study. An overall positive attitude towards CAM was observed among pharmacy students. Pharmacy students in the lower classes (first, second and third year classes) showed more positive attitudes toward CAM than their higher class counterparts (fourth and fifth year classes). However, no significant differences in sex, religion and age were observed. This positive attitude was demonstrated by the overwhelming endorsement of the need for CAM training at the undergraduate level at the Pharmacy faculty of COMAHS-USL as lack CAM knowledge and less access to evidence based scientific information were considered as common limitations to CAM practice. Future studies should look at student preferences with respect to appropriate learning methods, faculty members’ attitude and competency to teach CAM as well as the pattern of CAM use among patients and the public. These factors will inform and guide the development and implementation of CAM curricula that seeks to address the needs of an undergraduate Pharmacy student at COMAHS-USL.

## Electronic supplementary material

Additional file 1: **Survey Questionnaire.** (DOCX 23 KB)

## Abbreviations

CAM: Complementary and Alternative Medicine
COMAHS-USL: College of Medicine and Allied Health Sciences, University of Sierra Leone.
