# Supplementary material for: Awareness, use, attitude and perceived need for Complementary and Alternative Medicine (CAM) education among undergraduate pharmacy students in Sierra Leone: a descriptive cross-sectional survey
Source: BMC Complement Altern Med. 2014 Nov 8;14:438. doi: 10.1186/1472-6882-14-438 (PMC4236455; doi:10.1186/1472-6882-14-438)
Supplement: Supplementary file 1 — Additional file 1: Survey Questionnaire. (DOCX 23 KB) [file 12906_2014_2007_MOESM1_ESM.docx]

**Department of Pharmacognosy and Phytochemistry,**

**Faculty of Pharmaceutical Sciences**

**College of Medicine and Allied Health Sciences University of Sierra Leone.**

**Questionnaire**

**Awareness, Use, Attitude and Perceived Need for Complementary and Alternative Medicine (CAM) Among Undergraduate Pharmacy Students in Sierra Leone: A Cross-sectional Survey**

Dear Student,

We are conducting a survey of attitudes, awareness, and self-perceived perception skill on complementary and alternative medicine (CAM) use. Your input will be used to propose the development and implementation of CAM educational programs for students at COMAHS. Your responses will be anonymous and confidential and never associated with any information that could identify you personally. Only aggregated data from this survey will be reported. As there is no right or wrong answers to any item, please respond to each item according to how you feel about CAM at this point in time. Participation in this study is voluntary. You may decide not to complete this survey at any time without penalty.

Thank you.

**SECTION A: RESPONDENT DEMOGRAPHICS**

Please tick the most appropriate

**YEAR OF STUDY:** First Year Second Year Third Year Fourth Year

Fifth Year

**SEX:** Male Female

**Age Group:** 15-20yrs 21-26yrs 27-32yrs >33yrs

**Religion:** Christianity Islam

**SECTION B**

**AWARENESS AND USE OF CAM**

For each of the following 7 CAM modalities, please indicate (a) if you are aware of this modality; (b) have used or currently using this modality. **If your answer is yes, check the box--an unchecked box indicates an answer of no. Please check all that apply.**

| CAM Modalities | Awareness of CAM(a) | Use of CAM(b) |
| --- | --- | --- |
| Acupuncture |  |  |
| Herbal/Botanical/Supplements |  |  |
| Massage |  |  |
| Ayurveda |  |  |
| Spirituality/Prayer |  |  |
| Homeopathy |  |  |
| Meditation/Yoga/Relaxation |  |  |

If you have used any of the following above modalities, Please answer the following question

How effective do you think the modality/ies you have used is/are?

Very ineffective Ineffective Neutral

Effective Very effective

How harmful do you think the modality/ies you used is/are?

Very harmful Harmful, Neutral

Not harmful, Very not harmful

**SECTION C**

**Attitude towards Complementary and Alternative Medicine (CAM)**

Please read and respond to the following statements according to your beliefs, using the numbers 1-5 **where 1 is strongly disagree and 5 is Strongly agree**.

| Statements | Strongly disagree | Disagree | Neutral | Agree | Strongly agree |
| --- | --- | --- | --- | --- | --- |
| Clinical care should integrate best conventional and CAM practice |  |  |  |  |  |
| A patient's expectations, health beliefs and values should be integrated into the patient care process |  |  |  |  |  |
| Complementary therapies include ideas and methods from which conventional medicine could benefit. |  |  |  |  |  |
| Treatments not tested in a scientifically recognized manner should be discouraged |  |  |  |  |  |
| Complementary therapies are a threat to public health. |  |  |  |  |  |
| Health and disease are a reflection of balance between positive life-enhancing forces and negative destructive forces |  |  |  |  |  |
| Effects of complementary therapies are usually the result of a placebo effect |  |  |  |  |  |
| CAM treatment have no true impact on treatment of symptoms, disease conditions |  |  |  |  |  |
| Knowledge of CAM is important to me as a pharmacist |  |  |  |  |  |
| Health professional should be able advise patient on commonly used CAM methods |  |  |  |  |  |

**SECTION D**

**Sources of CAM Information**

Where do you obtain information and resources for evidence-based/educational materials on CAM? (Check all that apply.)

Books Media (Radio, TV, Newspaper) Journals

CAM practitioners Other Health professionals  Formal CAM Training

Training/Apprentice with healer

Perceived barriers to CAM implementation in Sierra Leone

Please tick the appropriate box

Lack of trained professionals YES NO

Lack of scientific evidence for Practice YES NO

Long time for treatment YES NO

Lack of Knowledge of CAM YES NO

**Perceived Need For Pharmacy Student For CAM Education.**

Do you think knowledge about CAM is useful to you as a future pharmacy professional?

YES NO

If NO, Can you please give reasons as to why ?…………………………………………………………………………..

…………………………………………………………………………………………………………………………………………………….

……………………………………………………………………………………………………………………………………………………

…………………………………………………………………………………………………………………………………………………….

……………………………………………………………………………………………………………………………………………………

Do you think CAM should be included into the Pharmacy undergraduate curriculum at COMAHS

**YES** **NO**

If **NO**, Can you please give reasons as to why? ………………………………………………………………………….

……………………………………………………………………………………………………………………………………………………

…………………………………………………………………………………………………………………………………………………….

…………………………………………………………………………………………………………………………………………………….

…………………………………………………………………………………………………………………………………………………….

***THANK YOU VERY MUCH FOR YOUR PARTICIPATION!***
